# Nasal carriage, risk factors and antimicrobial susceptibility pattern of methicillin resistant *Staphylococcus aureus* among healthcare workers in Adigrat and Wukro hospitals, Tigray, Northern Ethiopia

**DOI:** 10.1186/s13104-018-3353-2

**Published:** 2018-04-23

**Authors:** Haftom Legese, Atsebaha Gebrekidan Kahsay, Amlisha Kahsay, Tadele Araya, Gebre Adhanom, Saravanan Muthupandian, Araya Gebreyesus

**Affiliations:** 1 Department of Microbiology and Immunology, Institute of Biomedical Sciences, College of Health Science, Mekelle University, Mekelle, Ethiopia; 2 Department of Medical Laboratory, College of Medicine and Health Science, Adigrat University, Adigrat, Ethiopia

**Keywords:** Antimicrobial susceptibility test, Health care workers, methicillin resistance *Staphylococcus aureus*, nasal carriage, *Staphylococcus aureus*

## Abstract

**Objective:**

The aim of this study was to determine nasal carriage, risk factors and antimicrobial susceptibility pattern of methicillin resistant *Staphylococcus aureus* among health care-workers of Adigrat and Wukro hospitals Northern Ethiopia.

**Results:**

The overall prevalence of *S. aureus and* methicillin resistance *S. aureus* (MRSA) in the present study were 12% (29/242) and 5.8% (14/242) respectively. The rate of MRSA among *S. aureus* was 48.3%(14/29). In this study, MRSA carriage was particularly higher among nurse professionals (7.8%) and surgical ward (17.1%). None of the MRSA isolates were sensitive to penicillin and ampicillin. However, low resistance was found for chloramphenicol and clindamycin. Being diabetic and use of hands rub was statistically significant with MRSA colonization.

**Electronic supplementary material:**

The online version of this article (10.1186/s13104-018-3353-2) contains supplementary material, which is available to authorized users.

## Introduction

*Staphylococcus aureus* is known to be the cause of hospital and community acquired infections [1]. Methicillin resistant *S. aureus* (MRSA) causes a significant problem of the world and major health care associated pathogen [2, 3]. About 10–35% world population harbors MRSA in their anterior nares [4]. The emergence of MRSA is an important hospital acquired pathogen continues to remain a significant factor for failure of patient management worldwide [3–5].

Increasing rates of antibiotic resistance owing to an incautious use of antimicrobials lead to decrease treatment options for MRSA infection [6]. The increasing of MRSA strains becomes a public health problem [3]. This has a negative effect on the treatment cost, long hospitalization, and increased morbidity and mortality especially among the critically ill patients [7]. The problem of MRSA is observed all over the world, although, the burden of infection is high in developing countries [8].

High MRSA carriages of health care professionals have been reported as the key mechanism of transmission among patients during treatments, patients contact and aerosolization following sneezing [9]. Health care workers who have direct contact between the community and hospital may serve as the agents of the cross-transmission of the community acquired and hospital acquired MRSA [10].

Knowledge of MRSA prevalence and recent antimicrobial susceptibility pattern is very important for appropriate selection of the antimicrobial agents [11]. However, in most hospitals of African countries, there is neither surveillance system nor control policy for MRSA, this plays significant role for increasing the problem [12].

Therefore, this current study was aimed to determine nasal carriage, antimicrobial susceptibility patterns and associated factors of MRSA colonization among healthcare workers in Adigrat and Wukro hospitals, Tigray, northern Ethiopia. This evidence based information in the study area will contribute a role for the prevention and control of MRSA by responsible bodies.

## Main text

### Methods

#### Study area and study design

This study was carried out in Wukro and Adigrat general hospitals. Those hospitals are found in eastern zone of Tigray region and are located about 824 and 900 km respectively north of Addis Ababa (Capital city of Ethiopia). Wukro and Adigrat general hospitals have a total staffs 313 among those 41.3% are males and 58.7% are female, and are serve for the total population of 755,343. A cross sectional study was carried out among 242 health care workers from September to December 2016.

#### Isolation and identification

Swabs were inoculated on Manitol Salt agar (MSA) (Oxid, UK) and incubated at 37 °C for 24 h and sub cultured into blood agar. All positive culture was identified by their characteristics appearance and biochemical test using standard procedure. Colonies that were Manitol fermented (golden yellow colonies), β-hemolytic on blood agar were considered as *S. aureus* and was confirmed by Coagulase test as positive [13].

#### Antimicrobial susceptibility testing

Antimicrobial susceptibility testing was performed using modified Kirby–Bauer disc diffusion method on Muller–Hinton agar (MHA; Oxoid, UK) according Clinical and Laboratory Standards Institute (CLSI, 2016) guidelines [14]. From overnight grown colonies on nutrient agar 3–5 well-isolated colonies were emulsified in 3–4 ml of sterile physiological saline to get bacterial inoculums equivalent to 0.5 McFarland turbidity standards. After that the antibiotic discs were placed manually on the medium and incubated at 37 °C for about 18 h and the zones of inhibition was measured using caliper. The interpretation of the results was made based on the CLSI criteria as sensitive, intermediate and resistant [14]. Cefoxitin discs (30 μg), penicillin (10 μg), ampicillin (10 µg), erythromycin (15 µg), cotrimoxazol (25 µg), chloramphenicol (30 µg), gentamycin (10 µg), kanamycin (30 µg), amikacin (30 µg), ciprofloxacin (5 µg), tetracycline (30 µg), and clindamycin (2 µg) (Oxoid, UK). All isolates resistant to cefoxitin was considered as MRSA [14].

#### Data processing and analysis

The findings were statically analyzed using descriptive statistics, Chi square test (χ^2^) and p < 0.05 was considered as statistically significant. The variables from the demographic and associated risk analysis were performed using SPSS (version 22) package.

### Results

#### Socio-demographic characteristics

A total of 242 health professionals were included in the study. The age of study participants ranged from 20 to 59 years with mean age of 31.78 ± 8.9 years. One hundred forty-two (58.7%) were females and 100 (41.3%) were males. The mean number of their work experience was 9.1 years.

#### Prevalence of *Staphylococcus aureus* and *MRSA*

The prevalence of *S. aureus* and MRSA in this study was 12% (29/242) and 5.8% (14/242) respectively. The prevalence of MRSA among nurse, doctor and midwife professionals were 10 (7.8%), 1 (7.7%), and 2 (6.7%) respectively. The highest rate of *S. aureus* and MRSA observed in surgical ward were 7 (20.0%) and 6 (17.1%) respectively (Additional file 1: Table S1).

#### Risk factors associated for MRSA colonization

Chi square test (χ^2^) showed that use of hand rub (p < 0.001), and being a diabetic (p < 0.001), were statistically significant with MRSA colonization (Table 1).

**Table 1:** Risk factors associated with MRSA colonization among health professionals at Adigrat and Wukro hospitals, Tigray, Northern Ethiopia September–December 2016

Variable	MRSA		p value
	No, n (%)	Yes, n (%)	
Sex			
Male	93 (93)	7 (7.0)	0.497
Female	135 (95.1)	7 (4.9)	
Age group			
20–29	129 (94.2)	8 (5.8)	0.503
30–39	52 (91.2)	5 (8.8)	
40–49	30 (96.8)	1 (3.2)	
50–59	17 (100)	0 (0.0)	
Work experience			
< 5	110 (94)	7 (6.0)	0.486
6–10	56 (96.6)	2 (3.4)	
11–20	22 (88)	3 (12.0)	
21–30	40 (95.2)	2 (4.8)	
Department			
Medical	26 (96.3)	1 (3.7)	0.081
Surgical	29 (82.9)	6 (17.1)	
Pediatric	20 (90.9)	2 (9.1)	
Gynecology and obstetrics	28 (93.3)	2 (6.7)	
Laboratory	25 (100)	0 (0.0)	
Outpatient department	48 (96)	2 (4.0)	
Pharmacy	25 (96.2)	1 (3.8)	
Others	27 (100)	0 (0.0)	
Hand washing habit			
Always	116 (94.3)	7 (5.7)	0.298
Usually	91 (95.8)	4 (4.2)	
Rare	21 (87.5)	3 (12.5)	
Use of hand rub			
Always	123 (99.2)	1 (0.8)	0.001*
Usually	97 (95.1)	5 (4.9)	
Rare	8 (50)	8 (50)	
Prior hospitalization			
Yes	23 (88.5)	3 (11.5)	0.183
No	205 (94.9)	11 (5.1)	
History of antibiotics treatment			
Yes	115 (92)	10 (8)	0.127
No	113 (96.6)	4 (3.4)	
Chronic obstructive pulmonary disease			
Yes	28 (87.5)	4 (12.5)	0.081
No	200 (95.2)	10 (4.8)	
Diabetic mellitus			
Yes	7 (70)	3 (30.0)	0.001*
No	221 (95.3)	11 (4.7)	

*Statistically significant with MRSA colonization

NB: use of hand rub is use of a waterless alcohol [30]

#### Antimicrobial susceptibility patterns of *Staphylococcus aureus*

**Fig. 1:**
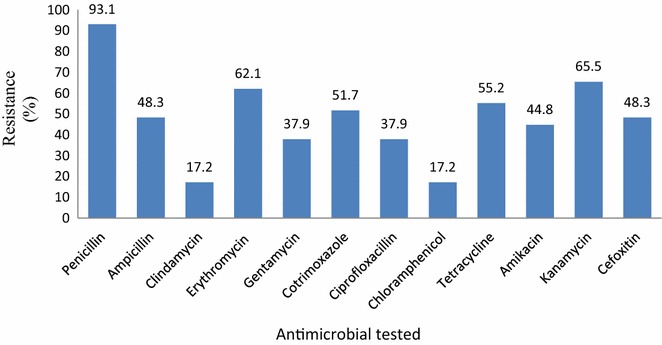
Antimicrobial susceptibility pattern of *S. aureus* strains to different antimicrobial agents at Adigrat and Wukro hospitals, Tigray, Northern Ethiopia September–December 2016 (n = 29)

The antimicrobial Susceptibility patterns were performed for the 29 *S. aureus* isolates against 12 antimicrobials. Of the 29 isolates, 93.1% showed resistance to penicillin followed by kanamycin 19 (65.5%), erythromycin 18 (62.1%), tetracycline 16 (55.2%) cotrimoxazole 15 (51.7%), ampicillin 14 (48.3%), and amikacin 13 (44.8%). Low resistance were found for chloramphenicol 5 (17.2%) and clindamycin 5 (17.2%). None of the isolates were intermediate resistance (Fig. 1).

#### Antimicrobial susceptibility pattern of methicillin resistance *S. aureus* (Additional file 2: Figure S1, Additional file 3: Table S2)

##### Multidrug resistance of *Staphylococcus aureus* isolates

According to Magiorakos et al. [15], multi-drug resistance in this study was considered as resistance to three or more of the antimicrobial class tested. Twenty-two (75.9%) of all the isolates were multi-drug resistant, five isolates were resistant for three and two isolates were resistant for ten antimicrobials (Table 2).

**Table 2:** Multi-drug resistance nature of *S. aureus* isolates at Adigrat and Wukro hospitals, Tigray, Northern Ethiopia September–December 2016

	Antibiotics	Number (%)
For three	PEN, AMP, CXT	2 (9.2%)
	PEN, ERY, TTC	1 (4.54%)
	PEN, ERY, AK	1 (4.54%)
	PEN, TTC, AK	1 (4.54%)
For four	PEN, ERY, TTC, AK	1 (4.54%)
	PEN, AMP, TTC, CXT	1 (4.54%)
	PEN, AMP, TTC, CXT	1 (4.54%)
For six	PEN, DA, TS, CIP, TTC, AK	1 (4.54%)
	PEN, GM, TS, CIP, TTC, AK	1 (4.54%)
	PEN, AMP, TS, TTC, AK, CXT	1 (4.54%)
For seven	PEN, DA, ERY, GM, TS, CIP, CHL	1 (4.54%)
	PEN, AMP, ERY, GM, TS, CIP, CXT	1 (4.54%)
For eight	PEN, ERY, GM, TS, CIP, CHL, TTC, AK	1 (4.54%)
	PEN, AMP, ERY, GM, TS, CIP, TTC, CXT	1 (4.54%)
	PEN, AMP, ERY, GM, TS, CHL, AK, CXT	1 (4.54%)
	PEN, AMP, DA, ERY, CIP, TTC, AK, CXT	1 (4.54%)
	PEN, AMP, ERY, GM, TS, CIP, TTC, CXT	1 (4.54%)
	PEN, AMP, ERY, GM, TS, TTC, AK, CXT	1 (4.54%)
	PEN, AMP, ERY, GM, TS, CIP, AK, CXT	1 (4.54%)
For ten	PEN, AMP, DA, ERY, GM, TS, CHL, TTC, AK, CXT	1 (4.54%)
	PEN, AMP, DA, ERY, GM, TS, CIP, CHL, TTC, CXT	1 (4.54%)
	Total	22 (100%)

*PEN* penicillin, *AMP* ampicillin, *GM* gentamycin, *AK* amikacin, *CHL* chloramphenicol, *CIP* ciprofloxacin, *TTC* tetracycline, *TS* cotrimoxazol, *DA* clindamycin, *ERY* erythromycin, *K* kanamycin, *CXT* cefoxitin

*MDR* multidrug resistant; MDR definition for *S. aureus* percent is computed from total number of *S. aureus*

### Discussion

The overall nasal carriage of *S. aureus* in the present study was 12%. This is supported by study carried out in India (14%) [10]. However, lower than that of reported from Ethiopia, (28.8%) [13], Democratic Republic Congo (16.5%) [16], Gaza Strip (31.1%) [17] Pakistan (48%) [18], China (25.3%) [19] and Iran (25.7%) [20].

The total prevalence of MRSA in this study was 5.78%. This was similar with results from [8], France (5.3%) [21], Asia (6.1%) [8] and Iran (5.3%) [20]. However, it was lower compared with the study revealed in Ethiopia, Mekelle (14.1%) [22] and Dessie (12.7%) [13], Egypt (20%) [23], Nigeria (39.9%) [12], Gaza Strip (25.5%) [17] and Pakistan (13.95%) [18]. On the other hand, our result was higher than study reported from and China (1.0%) [19]. This variations of prevalence among different study areas might be due to difference in rate of patient admission, study period [22], microbiological methods (from sample size to culture media) antimicrobial policy, in addition to that, variety levels of commitment to infection prevention measure among hospitals, and awareness of the health care worker about MRSA may contribute to the difference.

In current study, MRSA carriage was relatively higher among nurses (7.8%) followed by doctors (7.7%). This is consistent with study conducted in Ethiopia, Dessie [13], Gaza Strip [17] and India [10]. MRSA carriage was particularly high among surgical ward (17.1%) this result is comparable with corresponding study in Gaza Strip (35%) [17] and Dessie (35%) [13]. This result might be explained by the frequent direct physical contact of doctors and nurses with patients and increase workload in surgical wards.

In this study, use of hands was statistically significant with MRSA colonization. Health care workers rarely used hand rub were high proportion to have MRSA colonization on their anterior nare than those who were used hand rub usually and always. This finding is in line to previous studies in America [7], France [21], and Taiwan [24]. The temporary hand carriage of bacteria on the hands of health professionals could account for the major mechanism for the auto-transmission from contaminated hand to nose.

The present study, found that being diabetic patients was statistically associated with MRSA colonization. Health care workers with diabetic were high proportion to have MRSA colonization on their anterior nare. This was in line with studies from Tanzania [25] Iran [20], and Taiwan [26]. This may be due to diabetic patients reduced immunity which fails to combat the pathogens [25].

In the current study, there was no statistically significant of MRSA with educational status, hand washing habit, prior hospitalization, history of antibiotic treatment, and presence of chronic obstructive pulmonary disease in this study. This was in agreement with a result obtained in Ethiopia [13] and other studies conducted in other parts of the world [8, 20, 27].

Concerning antimicrobial susceptibility patterns of MRSA isolates, clindamycin and chloramphenicol were effective against MRSA isolates. However, increasing resistance was observed in our finding which is consistent with study reported from Pakistan ampicillin, penicillin, erythromycin, amikacin and ciprofloxacin (100%), (100%), (66%), (44%), and (33%) respectively [18]. Cotrimoxazole also showed a similar result compared with corresponding reports of Dessie (66.7%) [11]. Despite slight differences in the reported figures, the susceptibility patterns of antimicrobial were in line with the study from Nigeria for gentamycin 50 (63.3%), erythromycin 55 (69.6%) and Ciprofloxacin 32 (40.5%) [12], in India [3] for ciprofloxacin (34.6%) and erythromycin (54.8%), chloramphenicol (16.1%) from Serbia [27], and penicillin (93%) reported from India [10].

Higher susceptibility was also showed in the present study as compared to a result from health care workers at Iran for gentamycin (69%), clindamycin (69%), and ciprofloxacin (66%) [20]. Kanamycin also showed lower resistance compared with similar study in Serbia (90.3%) [27]. On the other hand, our finding was higher compared to studies conducted in India [28] ciprofloxacin (20%), and Gaza Strip erythromycin, tetracycline, gentamycin, clindamycin, and ciprofloxacin (19.6%), (9.8%), (3.9%), (3.92%), and (3.92%) respectively [17]. This resistance pattern of our finding might be due to excessive use of this antibiotics for many other infections and replacing of sensitive strains by resistance strains at the hospital settings.

Drug susceptibility test on all the 29 *S. aureus* isolates against 12 commonly used antibiotics were performed. The resistance of strains against penicillin, ciprofloxacin, and erythromycin is consistent with studies conducted in Ethiopia, Dessie [11], and Nepal [6], but cotrimoxazole (81.7%) and gentamycin (60.4%) were higher than our finding. However, lower resistance was observed with cotrimoxazole (33%), and gentamycin (27%) conducted in India [10], and 25% for gentamycin in Nepal [29]. This might be due to the variation in the geographical area, and local infection prevention and control strategies of the hospital settings.

Studies conducted in Ethiopia and China has reported higher resistance patterns to tetracycline (71.4%), chloramphenicol (57.1%) [11] and clindamycin (70%) [19]. Where as lower resistance than our finding to amikacin was reported from India [10], Nepal [6], and Pakistan [18]. In the present study higher resistance were showed for tetracycline, cotrimoxazol and gentamycin compared with study conducted in India [10]. In our study area, penicillin, ampicillin and erythromycin are the commonly prescribed antibiotics. This might have contributed for the resistance against these antimicrobials.

In this study high prevalence of multi drug resistance to wards *S. aureus* was observed. Of the total isolates 22 (75.9%) were resistant to three and above class of antimicrobials [15]. Fourteen of them (63.6%) were MRSA and comparable susceptibility was observed in a study from Ethiopia, Dessie [11]. This increased multi drug resistance might be due to continuous genetic variation of strains by mutation, or cross transmission of the a resistance genetic elements from one to another bacterium, overcrowded wards, and prescribed of antibiotics without culture and sensitivity [18].

## Conclusions

The present study, the overall prevalence of MRSA in the study area was found to be 5.78%. The carriage rate MRSA was worse among nurses and working in surgical wards. Rarely used hand rub and being diabetics were statistically significant with MRSA colonization. Clindamycin and chloramphenicol were sensitive antimicrobials for the treatment of MRSA and *S. aureus*. The majority of the *S. aureus* isolates were multidrug resistant.

## Limitation of the study

The infection is due to community or hospital acquired strains could not be identified. More sensitive and specific molecular techniques could not be used to identify the species and strain typing of *S. aureus*.

Furthermore, for the future researcher phenotypic and genotypic studies are needed to establish and clarify the genetic mechanism behind susceptibilities to antibiotics.

## Additional files

**Additional file 1: Table S1.** Prevalence of S*. aureus* and MRSA among health professionals in Adigrat and Wukro hospitals, Tigray, Northern Ethiopia September–December 2016.

**Additional file 2: Figure S1.** Antibiotic Susceptibility pattern of Methicillin Resistant *Staphylococcus aureus* strains to other antibiotics tested at Adigrat and Wukro hospitals, Tigray, Northern Ethiopia September–December 2016 (n = 14).

**Additional file 3: Table S2.** Antimicrobial susceptibility pattern of MRSA and MSSA isolates from health professionals at Adigrat and Wukro hospitals, Tigray, Northern Ethiopia September–December 2016.

## Abbreviations

AST: antimicrobial susceptibility testing
CLSI: Clinical and Laboratory Standards Institute
MDR: multi-drug resistance
MRSA: methicillin resistant *Staphylococcus aureus*
MSSA: methicillin sensitive *Staphylococcus aureus*
