# Supplementary material for: Nasal carriage, risk factors and antimicrobial susceptibility pattern of methicillin resistant Staphylococcus aureus among healthcare workers in Adigrat and Wukro hospitals, Tigray, Northern Ethiopia
Source: BMC Res Notes. 2018 Apr 23;11:250. doi: 10.1186/s13104-018-3353-2 (PMC5914064; doi:10.1186/s13104-018-3353-2)
Supplement: Supplementary file 1 — Additional file 1: Table S1. Prevalence of S. aureus and MRSA among health professionals in Adigrat and Wukro hospitals, Tigray, Northern Ethiopia September–December 2016. [file 13104_2018_3353_MOESM1_ESM.docx]

| **Variables** | | **No Sampled** | ***S.aureus***  **(n=29)**  **No (%)** | **MRSA**  **(n=14)** | |
| --- | --- | --- | --- | --- | --- |
|  |  |  |  | **No (%)** | **Total %(n=242)** |
| **Profession** | |  |  |  |  |
|  | Doctor | 13 | 2 (15.4) | 1 (7.7) | 1 (0.42) |
|  | Nurse | 129 | 16 (12.4) | 10 (7.8) | 10 (4.11) |
|  | Midwife | 30 | 6 (20.0) | 2 (6.7) | 2 (0.83) |
|  | Laboratory | 25 | 1 (4.0) | 0 (0.0) | 0 (0.0) |
|  | Others | 45 | 4 (8.9) | 1 (2.2) | 1 (0.42) |
| **Department** | |  |  |  |  |
|  | Medical | 27 | 3 (11.1) | 1 (3.7) | 1 (0.42) |
|  | Surgical | 35 | 7 (20.0) | 6 (17.1) | 6 (2.45) |
|  | Pediatric | 22 | 4 (18.2) | 2 (9.1) | 2 (0.83) |
|  | gyn & obs | 30 | 6 (20.0) | 2 (6.7) | 2 (0.83) |
|  | Laboratory | 25 | 1 (4.2) | 0 (0.0) | 0 (0.0) |
|  | Opd | 50 | 3 (6.0) | 2 (4.0) | 2 (0.83) |
|  | Pharmacy | 26 | 4 (15.4) | 1 (3.8) | 1 (0.42) |
|  | Others | 27 | 1 (3.6) | 0 (0.0) | 0 (0.0) |

***Key:** OPD = Outpatient Department; GYN &OBS=Gynecology and Obst
