# Supplementary material for: Nasal carriage, risk factors and antimicrobial susceptibility pattern of methicillin resistant Staphylococcus aureus among healthcare workers in Adigrat and Wukro hospitals, Tigray, Northern Ethiopia
Source: BMC Res Notes. 2018 Apr 23;11:250. doi: 10.1186/s13104-018-3353-2 (PMC5914064; doi:10.1186/s13104-018-3353-2)
Supplement: Supplementary file 2 — Additional file 2: Figure S1. Antibiotic Susceptibility pattern of Methicillin Resistant Staphylococcus aureus strains to other antibiotics tested at Adigrat and Wukro hospitals, Tigray, Northern Ethiopia September–December 2016 (n = 14). [file 13104_2018_3353_MOESM2_ESM.docx]

*Key: MRSA=Methicillin Resistant *Staphylococcus aureus*
