# Supplementary material for: Nasal carriage, risk factors and antimicrobial susceptibility pattern of methicillin resistant Staphylococcus aureus among healthcare workers in Adigrat and Wukro hospitals, Tigray, Northern Ethiopia
Source: BMC Res Notes. 2018 Apr 23;11:250. doi: 10.1186/s13104-018-3353-2 (PMC5914064; doi:10.1186/s13104-018-3353-2)
Supplement: Supplementary file 3 — Additional file 3: Table S2. Antimicrobial susceptibility pattern of MRSA and MSSA isolates from health professionals at Adigrat and Wukro hospitals, Tigray, Northern Ethiopia September–December 2016. [file 13104_2018_3353_MOESM3_ESM.docx]

| Antimicrobial | MRSA | |  | MSSA | |
| --- | --- | --- | --- | --- | --- |
|  | Sensitive N(%) | Resistant  N(%) |  | Sensitive  N (%) | Resistant  N (%) |
| Penicillin | 0(0.0) | 14(100.0) |  | 2(13.3) | 13(86.7) |
| Ampicillin | 0(0.0) | 14(100.0) |  | 15(100.0) | 0(0.0) |
| Clindamycin | 11(78.6) | 3(21.4) |  | 13(86.7) | 2(13.3) |
| Erythomycin | 5(35.7) | 9(64.3) |  | 6(40.0) | 9(60.0) |
| Gentamycin | 6(42.9) | 8(57.1) |  | 12(80.0) | 3(20.0) |
| Cotrimexazole | 5(35.7) | 9(64.3) |  | 9(60.0) | 6(40.0) |
| Ciprofloxacillin | 8(57.1) | 6(42.9) |  | 10(66.7) | 5(33.3) |
| Chloramphenicol | 11(78.6) | 3(21.4) |  | 13(86.7) | 2(13.3) |
| Tetracycline | 5(35.7) | 9(64.3) |  | 8(53.3) | 7(46.7) |
| Amikacin | 7(50.0) | 7(50.0) |  | 9(60.0) | 6(40.0) |
| Kanamycin | 3(21.4) | 11(78.6) |  | 7(46.7) | 8(53.3) |
| Cefoxitin | 0(0.0) | 14(100.0) |  | 15(100.0) | 0(0.0) |

**Key:** * MRSA, methicillin-resistant *Staphylococcus aureus*; * MSSA, methicillin-sensitive *Staphylococcus aureus*
